# Study on the enhancement method of online monitoring image of dense fog environment with power lines in smart city

**DOI:** 10.3389/fnbot.2022.1104559

**Published:** 2023-01-06

**Authors:** Meng Zhang, Zhitao Song, Jianfei Yang, Mingliang Gao, Yuanchao Hu, Chi Yuan, Zhipeng Jiang, Wei Cheng

**Affiliations:** ^1^State Grid Hubei Power Supply Limited Company Ezhou Power Supply Company, Ezhou, China; ^2^School of Electrical and Electronic Engineering, Shandong University of Technology, Zibo, China

**Keywords:** smart city, power lines, atmospheric scattering model, global atmospheric light, dark pixels

## Abstract

In this research, an image defogging algorithm is proposed for the electricity transmission line monitoring system in the smart city. The electricity transmission line image is typically situated in the top part of the image which is rather thin in size. Because the electricity transmission line is situated outside, there is frequently a sizable amount of sky in the backdrop. Firstly, an optimized quadtree segmentation method for calculating global atmospheric light is proposed, which gives higher weight to the upper part of the image with the sky region. This prevents interference from bright objects on the ground and guarantees that the global atmospheric light is computed in the top section of the image with the sky region. Secondly, a method of transmission calculation based on dark pixels is introduced. Finally, a detail sharpening post-processing based on visibility level and air light level is introduced to enhance the detail level of electricity transmission lines in the defogging image. Experimental results indicate that the algorithm performs well in enhancing the image details, preventing image distortion and avoiding image oversaturation.

## 1. Introduction

With the development of the Internet of Things sensor and image processing technology, the monitoring requirements of the power system for the transmission line are gradually improved. The transmission line can be equipped with image sensors to observe its running status in real time, which leads to potential risks of prefabrication. As an important part of power system, electricity transmission line is an important way of power resource transmission. Its operation stability will have a direct impact on power quality. People are always concerned with monitoring electricity transmission lines in order to guarantee the secure and reliable operation of those lines. Electricity transmission lines are installed outdoors and exposed to fog, rain, dew and other weather conditions for a long time, and are greatly affected by the environment. Faults such as insulator defects may occur, which will seriously affect the normal use of the electricity transmission line and reduce the service life of the line. Once the electricity transmission line fails, accidents such as tripping and power outages may occur, resulting in human and economic losses. Therefore, regular inspection of the electricity transmission line is of great significance to ensure the reliable, safe, and efficient operation of the electricity transmission line. The inspection of electricity transmission lines has been dominated by manual inspection for a long time, but manual inspection requires staff to work in the outdoor environment for a long time, which not only has poor monitoring efficiency and accuracy, but also has potential safety hazards for staff. Therefore, in recent years, the method of monitoring electricity transmission lines through a video monitor system has been widely used, which is of importance to improve the monitoring efficiency of electricity transmission lines and speed up the construction of smart cities.

In recent years, constant fog has become one of the terrible weather situations damaging the power grid’s atmospheric environment as a result of the rapid development of the economic scale and the acceleration of urbanization. Fog is a common occurrence in the atmosphere. In foggy circumstances, the air is dense with atmospheric particles that not only absorb and scatter the reflected light from the scene, but also disseminate some of it into the observation equipment (Xu et al., 2015). Therefore, in haze weather, the images obtained by the monitoring system and the vision system will be seriously degraded, such as image color offset, reduced visibility, loss of details, and other problems, which seriously affect image detection, tracking, recognition and the use of the monitoring system (Su et al., 2020). Electricity transmission line monitoring in hazy weather will face some problems, such as reduced contrast, chromatic aberration, and unclear details, which will significantly impact the visual impact of monitoring power transmission lines, adversely affect transmission line monitoring, and even cause misjudgment. Therefore, it is necessary to conduct defogging research for transmission line monitoring.

The two kinds of defogging algorithms that are now most often utilized are image enhancement and image restoration. Since computer hardware has improved quickly in recent years, image defogging algorithms based on machine learning have also been proposed (Sharma et al., 2021). The image enhancement-based defogging algorithm merely improves the image contrast and other characteristics using image enhancement technology to achieve the defogging goal. It does not take into account the physical process of fog generation. Traditional image contrast enhancement methods include histogram redistribution (Zhou et al., 2016), intensity transformation (Sangeetha and Anusudha, 2017), homomorphic filtering (Seow and Asari, 2006), wavelet transform (Jun and Rong, 2013), and Retinex algorithm (Jobson et al., 1997b). In Retinex theory, the image is made up of the incident element which represents the brightness information around the object and the reflection element which reflects the reflection ability of itself, then the single scale Retinex algorithm (SSR) is proposed. And then, multiscale Retinex with color restoration (MSRCR) and the multiscale Retinex (MSR) algorithm have both been developed on the foundation of SSR (Jobson et al., 1997a). Defogging algorithm based on image restoration is more commonly used at present. Such algorithms need to consider the physical processes of fog formation, and reasonably estimate the transmission and atmospheric light. In the end, the atmospheric scattering model’s calculations provide the restored image. Please note that the word “transmission” mentioned here is not the same as the word “transmission” in the electricity transmission line mentioned above. The “transmission” mentioned here is a parameter in the atmospheric scattering model that reflects the distance between the object in the image and the observation point (such as the camera). Without special circumstances, the t appears later to refer to transmission in atmospheric scattering models.

Multiple image defogging is mainly based on polarization method. Schechner proposed a method of defogging by using two polarized images taken vertically and horizontally (Schechner et al., 2001). Miyazaki et al. (2013) suggested a fog removal method based on the polarization data of two known photographs taken at various distances to predict the characteristics of fog. Shwartz and Schechner (2006) suggested a polarization defogging technique for images without sky areas that choose two comparable characteristics in the scene to estimate atmospheric scattering model parameters. However, the polarization-based image defogging algorithm needs to take multiple polarized images in the same weather condition, which is hard to fulfill the practical needs.

Due to the large limitations of multiple image defogging, it has not been widely used. The more commonly used defogging method is the restoration-based single image defogging method. To estimate necessary parameters based on atmospheric scattering model, Fattal (2008) created the concept of surface shading and the assumption that the transmission and surface shadow are unrelated. Based on the supposition that fog-covered images have less contrast than those taken in clear skies, Tan (2008) proposed an defogging algorithm for images based on the Markov random field optimization atmospheric scattering model to maximize local contrast. Meng et al. (2013) offered a technique to calculate the transmission of unknown scenes by combining the boundary constraint of single image defogging with context regularization based on weighted L1 norm. He et al. (2010) proposed a defogging algorithm called dark channel prior. For single image defogging, the dark channel prior algorithm has developed as one of the most popular methods. In order to reduce halo and block artifacts generated by coarse transmission estimation, He uses “soft matting” to smooth up the coarse transmission. However, the soft matting technique has the disadvantage of consuming too much time, so it is hard to apply in actual situations. To resolve this issue, He et al. (2012) proposed a guide filter and a fast guide filter (He and Sun, 2015). The neighborhood pixels relationship of hazy images may be transferred by the guided filter to improve air light and transmission smoothness. However, dark channel prior algorithm has some limitations. Dark channel prior algorithm is ineffective for sky region or bright ground region, and the result of defogging in this region is often oversaturated. And dark channel prior algorithm is poor in the processing of depth discontinuous region, and in the area where the foreground and background of the image meet, “halo” phenomena are simple to create. Tarel and Hautiere (2009) proposed a median filter and its variants to replace soft matting, which can improve the calculation speed. Ehsan et al. (2021) proposed a fog removal method that uses local patches of different sizes to calculate the two transmission maps and refine the transmission map with gradient-domain guided image filtering. With the help of training the sum of squared residual error, Raikwar and Tapaswi (2020) suggested a method to determine the lower limit of transmission based on the peak signal-to-noise ratio. Berman and Avidan (2016) assumed that an image can be approximated by hundreds of different colors, which form close clusters in RGB space, and thus proposed a non-local prior method of defogging.

More and more fog removal algorithms based on machine learning have been presented as a result of the advancement of computer neural networks and deep learning. Li et al. (2017) reconstructed the atmospheric scattering model. Then, to estimate the pertinent parameters of fog, an All-in-One Dehazing Network was created utilizing residual learning and convolutional neural network. GridDehazeNet is Liu’s proposed end-to-end trainable convolutional neural network for removing fog from a single image. It has pre-processing, backbone, and post-processing, it is a multi-scale network image defogging algorithm based on attention (Liu et al., 2019). Cai et al. (2016) proposed a deep CNN structure for fog removal, named Dehaze Net, to achieve end-to-end fog removal. Zhang and Patel (2018) proposed an edge-preserving densely connected encoder-decoder structure fusion end-to-end densely connected pyramid defogging network, named DCPDN. Pang et al. (2020) suggested a binocular image dehazing Network, which requires the simultaneous use of multiple images for defogging. Ren et al. (2018) suggested a Gated Fusion Network for image defogging, which fuses the three inputs preprocessed for foggy images to avoid halo artifacts. Qin et al. (2020) proposed an attention-based feature fusion single image dehazing network, named FFA-Net.

In order to promote the construction of smart cities, we propose a defogging algorithm for electricity transmission line monitoring. The following are the paper’s contributions:

• In order to solve the problem of inaccurate calculation of global atmospheric light in the original dark channel prior algorithm, according to the assumption that the sky area of the electricity transmission line image is usually in the upper half of the image, an improved quadtree segmentation is proposed to calculate the global atmospheric light value. The algorithm can avoid the interference caused by the bright objects on the ground to the solution of the global atmospheric light;
• The concept of dark pixel is introduced for the problem that the dark channel prior is prone to the “halo” effect. Dark pixels are located using super pixel segmentation and a fidelity function is proposed to calculate the transmission;
• Due to the size of the electricity transmission line in the image is tiny and difficult to observe, a detail sharpening post-processing based on visibility and air light is introduced to improve the image details of the electricity transmission line.

The remainder of this paper is organized as shown below. (Section “2 Related works) reviews atmospheric scattering models and dark channel priors, and points out the limitations of dark channel priors. (Section “3 Proposed method) presents a defogging method for electricity transmission line images based on improved quadtree segmentation and dark pixels, and enhances image details. (Section “4 Experimental results and discussion) evaluates the efficacy of the proposed method using both qualitative and quantitative analyses. And the entire study is summarized in (Section “5 Conclusion).

## 2. Related works

### 2.1. Physical model

The physical model of atmospheric scattering based on Mie scattering theory was initially put out by McCartney (1976). Narasimhan and Nayar (2001) believes that the wavelength of visible light in a uniform atmosphere has nothing to do with the scattering coefficient, and proposed a simplified version of the atmospheric scattering model:


(1) $$I\left(x\right)=I_{\infty }\rho \left(x\right)e^{-\beta d\left(x\right)}+I_{\infty }\left(1-e^{-\beta d\left(x\right)}\right)$$

**FIGURE 1:**
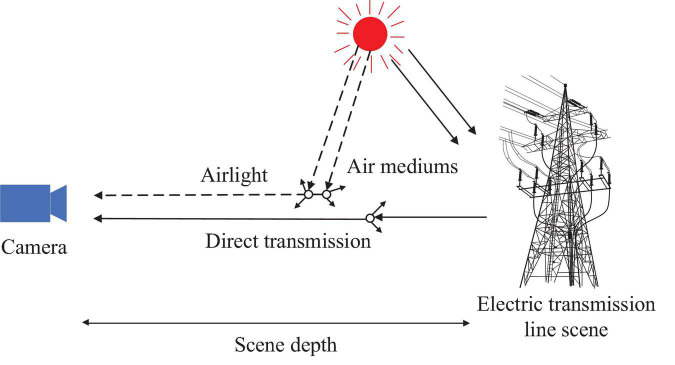
Atmospheric scattering model.

In formula (1), I is the brightness of the sky, *ρ*(*x*) denotes the normalized radiance of a scene point *x*, *β* is the scattering coefficient of the atmosphere, and *d* is the scene depth. However, this model is too complicated, so a simplified atmospheric scattering model is proposed. The simplified atmospheric scattering model developed by he is now the most used atmospheric scattering model for expressing the principle of fog (He et al., 2010). It is shown in the following formula:


(2) I(x)=J(x)t(x)+A(1-t(x))

where *A* is the global atmospheric light, which represents the background lighting in the atmosphere, and *I*(*x*) and *J*(*x*) are the fogging and defogging images, respectively. And *x* = (*m, n*) is the coordinate of the image. *t*(*x*) is transmission. It represent the transmission of a medium that is not scattered and successfully entries into vision systems such as monitoring systems and cameras. As per the atmospheric scattering theory, the scattering of air light during the process of reaching the vision system and the attenuation process of the reflected light from the surface of the object reaching the vision system are the two main divisions of the scattering of atmospheric particles. For equation (2), *J*(*x*)*t*(*x*) is direct transmission, and *A*[1- *t*(*x*)] is airlight, denoted as *a*(*x*). Direct transmission means the attenuation of the foggy image directly passing through the air medium, and the airlight is generated by the scattered light. The schematic diagram for the atmospheric scattering model is shown in Figure 1. Note that the solid line represents direct transmission and the dashed line represents airlight.

For transmission *t*(*x*), we have:


(3) $$t\left(x\right)=e^{-\beta d\left(x\right)}$$

In the above formula, *d*(*x*) represents the scene depth and, at the same time, *β* is the atmospheric scattering coefficient. The formula shows that the transmission decreases gradually as the depth of the scene increases.

Trying to imply the transmission *t*(*x*) and the global atmospheric light value *A* into the atmospheric scattering physical model yields the defogging image *J*, which is the essential step in image defogging based on the atmospheric scattering model. The following formula can be obtained by deriving formula (2):


(4) $$J\left(x\right)=\frac{I\left(x\right)-A}{t\left(x\right)}+A\left(x\right)$$

It can be seen from formula (4) that the key to calculating the defogging image is to reasonably estimate the transmission *t*(*x*) of the foggy image and the global atmospheric light value *A*. At present, the most commonly used method of defogging is the dark channel prior theory proposed by He et al. (2010).

### 2.2. Dark channel prior theory

He gained a statistical rule by observing a significant number of images without fog: for a large number of non-sky local patches, there is always at least one color channel with pixel intensity so low that it is close to 0. So the dark channel *J^dark^*(*x*) is defined by the following formula:


(5) $$J^{dark}\left(x\right)=\min_{y\in \Omega \left(x\right)}\left\{\min_{c\in \left\{r,g,b\right\}}\left[J^{c}\left(y\right)\right]\right\}$$

where Ω(*x*) is the local area centered at *x, y* is the pixel in the local area Ω(*x*), *J^C^* is the color channel of the fog-free image *J, C* is the three channels of the RGB image. And *r, g, b* represent the red, green and blue channels of the RGB image, respectively.

He draws the following conclusion through observation: for an outdoor fog-free image *J*, due to the shadows caused by buildings in the city or leaves in the natural landscape, the surfaces of colored objects with low reflectivity, and the surfaces of dark objects, dark channel intensity of *J* for non-sky regions is exceedingly low, almost nothing. So there is the following formula:


(6) $$J^{dark}\to 0$$

The transmission calculation formula may be constructed using the dark channel prior theory and the atmospheric scattering model above as follows:


(7) $$t\left(x\right)=1-\omega \min_{y\in \Omega \left(x\right)}\left\{\min_{c}\left[\frac{I^{c}\left(y\right)}{A^{c}}\right]\right\}$$

The role of *ω* is to retain some fog to make the image appear more natural. The value range of *ω* is (0, 1), and the value is generally set to 0.95.

The transmission obtained by this method is not accurate, and is prone to “halo” effect. The “halo” phenomenon is an effect that tends to occur in images after the fog has been removed. Since the foreground is close to the observation point and the background is far from the observation point in the image, the depth of field of different positions in the image has a large gap, especially for the junction of the foreground and background. Therefore, the “halo” phenomenon is usually generated at the junction of the foreground and background of the image, resulting in abnormal color distortion at the edge of the observed object in the image after fog removal, and the “halo” phenomenon gradually weakens when the image is far away from the edge. Therefore, He optimized the transmission using “soft matting” to get rid of the “halo” effect. However, the “soft matting” consumes a lot of time, so it is not suitable or practical applications. Therefore, He proposed the guided filter, through which the transmission optimization time can be greatly shortened, and the resulting image edges are sharper.

In order to prevent the image from being enhanced too much due to too small transmission, it is required to define the bottom bound of transmission *t*_0_, which is usually set to 0.1. Then the final result can be obtained from the following equation:


(8) $$J\left(x\right)=\frac{I\left(x\right)-A}{\max\left(t\left(x\right),t_{0}\right)}+A$$

It can be seen from Formula (8) that for a given fogged image *I*[*x*], to obtain the image after defogging [that is, *J*(*x*)], only two unknown quantities need to be solved: global atmospheric light value *A* and transmission t. Therefore, when using the atmospheric scattering model for image defogging, the most important two steps are the calculation of global atmospheric light *A* and the calculation of transmission *t*.

### 2.3. Disadvantages of dark channel priors

In the dark channel prior algorithm, the global atmospheric light is chosen in the brightest color channel in the image. He picks the pixels with the highest intensity as the global atmospheric light after first detecting the brightest top 0.1 percent of the dark channel pixels. However, this process suffers from large areas of white objects or objects that are too bright in the image. At this point the global atmospheric light is misestimated, resulting in a color shift in the recovered image. Second, it is common to create a “halo” phenomenon in the region separating the image’s foreground and background when employing the dark channel prior algorithm for regions with discontinuous depths. Finally, the atmospheric scattering in the real situation is multiple scattering. The single scattering model is the most often used atmospheric scattering model since it is challenging to compute multiple atmospheric scattering. As a result, the defogging images obtained by the dark channel prior algorithm are often too smooth and lack of image details. Therefore, this paper will optimize the dark channel prior algorithm for these three aspects.

## 3. Proposed method

**FIGURE 2:**
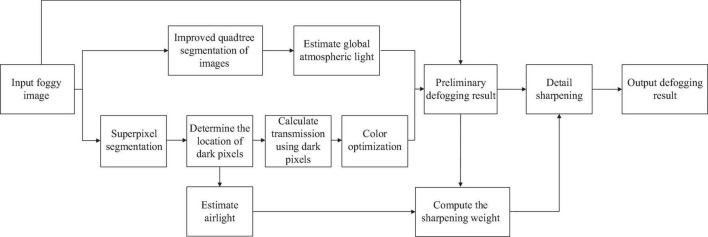
The flow chart of the proposed method.

The defogging algorithm flowchart from this work is shown in Figure 2. Electricity transmission lines and power towers are often located outdoors, and their images often have large areas of the sky. According to the statistical law that the sky area often exists in the upper part of the image, in order to address the issue of erroneous estimation of the global atmospheric light due to the influence of a large area of white objects, a global atmospheric light solution based on the optimized quadtree algorithm is proposed. This ensures correct estimation of global atmospheric light. Then we define dark pixels, perform superpixel segmentation on the input foggy image, and locate dark pixels in the segmented superpixel block. The transmission is calculated through a fidelity function, and the solved transmission is optimized for color correction. The next step is to invert the atmospheric scattering model to produce a preliminary defogging image. Due to the thin size of electricity transmission line, which is not suitable for observation, and the defogging image lacks details, a detail sharpening post-processing algorithm based on airlight constraints and visibility constraints are used for the preliminary defogging image to improve the texture details of the image. Finally, the final defogging image *J*(x) is obtained.

### 3.1. Global atmospheric light estimation

The presence of fog in the image will lead to a brighter area in the image, so the global atmospheric light is usually selected in the bright area, usually in the sky area. However, white objects on the ground or high-brightness objects can easily induce an incorrect selection of the global atmospheric light, resulting in chromatic aberration in the fog-free image. Therefore, it is necessary to improve the selection of global atmospheric light.

An optimized quadtree segmentation algorithm is used to estimate global atmospheric light in this study. Based on the principle that the pixel value variance of the image is often low in the foggy area, Kim et al. (2013) suggested an algorithm based on quadtree segmentation to select the global atmospheric light. The input image is first divided into four sections. Then we name the upper left area as area A, the upper right area as area B, the lower left area as area C, and the lower right area as area D. Then, for each region, we determine the standard deviation and mean of the pixel values for the three R, G, and B channels, and subtract the standard deviation from the mean to obtain a region score. The region with the highest score is first determined. It is then divided into four smaller parts, and the region with the highest score is selected from those four. Repeat the aforementioned procedure up until the size of the selected area is below the predetermined threshold. In the final selected area, we look for the value of the pixel closest to the white area as the global atmospheric light. For an RGB image, the white part is the area where the three channels of R, G, and B are all 255. Hence the estimate of global atmospheric light can be transformed into finding the minimum of the following formula:


(9) $$||\left(I_{r}\left(x\right),I_{g}\left(x\right),I_{b}\left(x\right)\right)-\left(255,255,255\right)||$$

**FIGURE 3:**
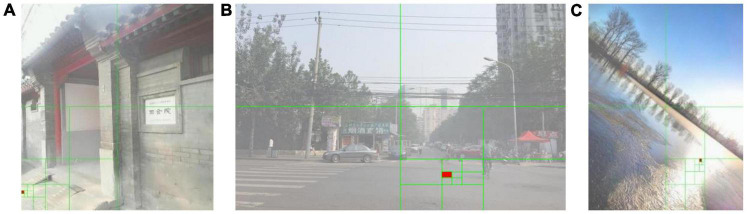
Estimation of global atmospheric light using quadtree segmentation algorithm.

In Formula (9), *I* represents the input image, *r, g, b* represent the RGB color channel of the input image, *x* represents the pixel value of each pixel of the input image, and 255 represents the white in the RGB space. Through the quadtree segmentation method, the global atmospheric light can be selected in a brighter area as much as possible. However, when there are large areas of white objects or high-contrast objects in the non-sky area, the quadtree segmentation algorithm will still select the non-sky area as the global atmospheric light, as shown in Figure 3. Images are from the OTS dataset in the Realistic Single Image Dehazing dataset. We usually call it RESIDE. The green line in the image represents the quadtree segmentation process, and the red fill represents the final selected area. It can be seen from Figure 3 that the estimation of the global atmospheric light may be disturbed by the white objects on the ground, and the final result is chosen on the ground and the lake surface instead of the sky.

**FIGURE 4:**
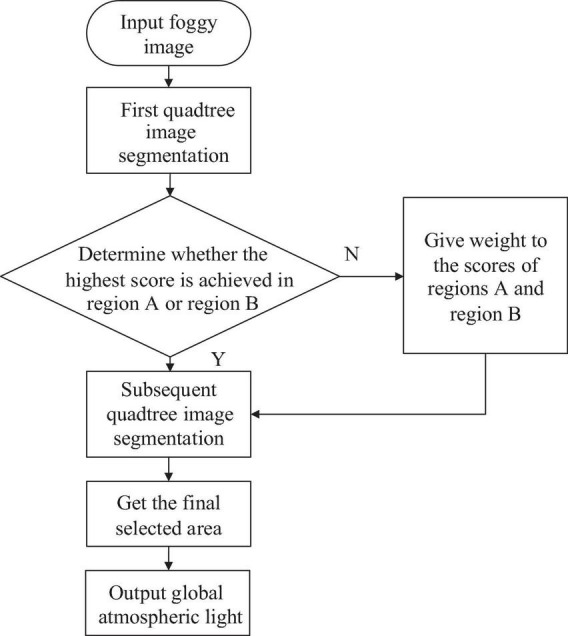
Flowchart of the optimized quadtree segmentation algorithm.

We optimize the quadtree segmentation for estimating the global atmospheric light to address this issue. For the four regions A, B, C, and D, we calculate their regional scores and record them as *score*_*A*_, *score*_*B*_, *score*_*C*_, and *score*_*D*_, and then compare the scores. Because the sky is mostly concentrated in the upper portion of the image, if the area with the highest score in the first step is located in the upper half of the image, that is, the *score*_*A*_ or the *score*_*B*_ has the highest score, the subsequent segmentation operation will be continued. If the region with the highest score in the first step is located in the lower half of the image, that is, the *score*_*C*_ or *score*_*D*_ has the highest score, then we assign the calculation weights *ξ_*A*_* and *ξ_*B*_* to the *score*_*A*_ and *score_*B*_*, respectively, and the scores are recorded as *ξ_*A*_*⋅*score*_*A*_ and *ξ_*B*_*⋅*score*_*B*_. Then calculation process returns to the first segmentation process, and re-compare the scores of *ξ_*A*_*⋅*score*_*A*_, *ξ_*B*_*⋅*score*_*B*_, *score*_*C*_, and *score*_*D*_, so that the global atmospheric light can be located at the top half of the image in the first step. To ensure that the recalculated *ξ_*A*_*⋅*score*_*A*_ and *ξ_*B*_*⋅*score*_*B*_ can be larger than *score*_*C*_ and *score*_*D*_, the calculation weight *ξ* is set to 1.5. Finally, the average value of the selected area is used as the global atmospheric light. Figure 4 depicts the process mentioned previously.

**FIGURE 5:**
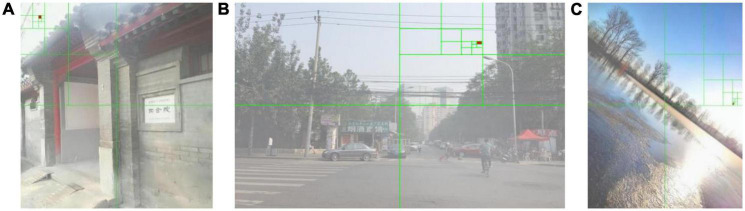
Estimation of global atmospheric light using an optimized quadtree segmentation algorithm.

Figure 5 shows the global atmospheric light selection result for the three foggy images given in Figure 3 using the optimized quadtree segmentation algorithm. The selected area of the global atmospheric light is changed from bottom half of images to the sky area. This shows that for foggy images with a sky, this method can locate the global atmospheric light in the sky area in the upper half of the image, and avoid locating it on the ground or large areas of white objects and other interfering objects.

### 3.2. Transmission optimization

Since the dark channel prior uses the minimum filter to calculate the transmission, it is common for a depth discontinuity to emerge at the image’s boundaries. Hence it is easy to produce a “halo” phenomenon at the boundary between the foreground and background. When the foggy image is converted into a dark channel map to extract the clear part, the size of the local patch Ω is difficult to determine. When deriving the transmission calculation formula, the original dark channel prior method assumes that the transmission in the local patch Ω is a constant, which is not consistent with the real situation. To optimize transmission, a method combining super-pixel segmentation and the dark pixel is utilized in this research.

Zhu pointed out in Zhu et al. (2019) that dark pixels are widespread. First, this paper defines dark pixels as follows:


(10) $$\min_{c}J^{c}\left(z\right)\to 0$$

Simple linear iterative clustering (Achanta et al., 2012) is used for super-pixel segmentation of images. The technology can be called SLIC for short. Superpixel segmentation uses adjacent pixels with the same brightness and texture characteristics to form irregular pixel blocks, and aggregates pixels with similar characteristics to achieve the purpose of using a small number of superpixel blocks to replace a large number of pixels in original images. When the superpixel block is too large, block artifacts and “halo” phenomena may also occur, which are caused by the discontinuity of depth caused by the excessively large superpixel, so the size of the superpixel block needs to be selected reasonably. We partition the foggy image into 1,000 superpixels in this study. Next, we need to locate dark pixels in the generated superpixel block. We locate dark pixels using the local constant assumption (Zhu et al., 2019). Note that the local constant assumption is only used to locate dark pixels, not to estimate transmission. For each superpixel local patch Ω, there is at least one dark pixel in it. From the assumption that the amount of transmission in the local patch Ω of each superpixel is constant, it can be known that dark pixels are found in each superpixel local area Ω by finding a local minimum in ${\text{mi}n}_{c}I_{w}^{c}$, where $\min_{c}I_{w}^{c}=\min_{c}\text{[}I^{c}\left(x\right)A^{c}]$.

For each dark pixel, there is the following formula:


(11) $$\min_{c}\frac{I^{c}\left(z\right)}{A^{c}}=\left[1+t\left(z\right)\right]-t\left(z\right)\min_{c}\frac{J^{c}\left(z\right)}{A^{c}}$$

A certain amount of fog should be preserved in order to give the image a more realistic appearance, and take J^c^ (z)/A^c^ = 0.05 (Zhu et al., 2019). Bringing formula (10) into formula (11), there is the following formula:


(12) $$0.95t\left(z\right)\approx 1-\min_{c}\frac{I^{c}\left(z\right)}{A^{c}}$$

For any pixel *x*, the smaller the value of min_*c*_ [*I^c^* (*x*)/*A^c^*] = min_Ω,*c*_ [*I^c^* (*x*)/*A^c^*] is, the closer min_*c*_ [*I^c^* (*x*)/*A^c^*] is to the minimum value of the local patch Ω, and the more likely the pixel is to be a dark pixel (Zhu et al., 2019). To ensure that *x* is a dark pixel, min_*c*_ [*I^c^* (*x*)/*A^c^*] and min_Ω,*c*_ [*I^c^* (*x*)/*A^c^*] should be close enough. Therefore we define the fidelity function *F*(*x*) for the dark pixel *x* as follows:


(13) $$F\left(x\right)=\log_{0.001}\left\{\max\left[\min_{c}\frac{I^{c}\left(x\right)}{A^{c}}-\min_{\Omega ,c}\frac{I^{c}\left(x\right)}{A^{c}},0.001\right]\right\}$$

As can be seen from the above, the closer min_*c*_ [*I^c^* (*x*)/*A^c^*] and min_Ω,*c*_ [*I^c^* (*x*)/*A^c^*] are, the more likely pixel *x* is to be a dark pixel. Formula (13) is a fidelity function. According to this formula, when the difference between min_*c*_ [*I^c^* (*x*)/*A^c^*] and min_Ω,*c*_ [*I^c^* (*x*)/*A^c^*] is less than 0.001, it can be seen from the property of logarithmic function that the value of *F*(*x*) is 1, thus min_*c*_ [*I^c^* (*x*)/*A^c^*] = min_Ω,c_ [*I^c^* (*x*)/*A^c^*]. Therefore, it can be approximately considered that the pixel *x* is the expected dark pixel. Therefore, there is the following formula:


(14) $$\tilde{t}\left(x\right)\approx \frac{\left[1-\min_{c}\frac{I^{c}\left(x\right)}{A^{c}}\right]}{0.95}=\frac{1-\min_{\Omega }\left[\min_{c}\frac{I^{c}\left(x\right)}{A^{c}}\right]}{0.95}$$

The final transmission is obtained by optimizing the following energy function:


(15) $$E\left(t\right)=\sum_{x}F\left(x\right){\left[t\left(x\right)-\tilde{t}\left(x\right)\right]}^{2}+\lambda \left[a_{x,N\left(\tilde{t}\right)}{\left(\frac{\partial t}{\partial x}\right)}^{2}+a_{y,N\left(\tilde{t}\right)}{\left(\frac{\partial t}{\partial y}\right)}^{2}\right]$$

where $a_{x,N\left(\tilde{t}\right)}$ and $a_{y,N\left(\tilde{t}\right)}$ are weight coefficients, defined as:


(16) $$a_{x,N\left(\tilde{t}\right)}={\left[{\left|\frac{\partial \left(I^{c}/A^{c}\right)}{\partial x}\right|}^{2}+\varepsilon \right]}^{-1}$$

(17) $$a_{y,N\left(\tilde{t}\right)}={\left[{\left|\frac{\partial \left(I^{c}/A^{c}\right)}{\partial y}\right|}^{2}+\varepsilon \right]}^{-1}$$

The final transmission can be obtained from the above formula, as shown in the following formula:


(18) $$\vec{t}={\left(\vec{F}+\lambda \vec{L}\right)}^{-1}\vec{F}\vec{\tilde{t}}$$

where $\vec{t}$ is the vector form of *t*, $\vec{\tilde{t}}$ is the vector form of $\tilde{t}$. And $\vec{F}$ is a sparse diagonal matrix composed of elements in *F*, $\vec{L}$ is the Laplace matrix. The value of *λ* is 0.02.

The restored fog-free image may have color offset problems such as too dark color in non-sky area and overexposure color in bright sky area. It is necessary to perform color correction on the obtained transmission. Color correction for transmission *t* is performed by the following formula:


(19) $$t=\frac{\max\left(t,1-\min_{c}\frac{I^{c}\left(x\right)}{A^{c}}\right)+\sigma }{1+\sigma }$$

where 0.2 is used as the value for *σ*.

**FIGURE 6:**
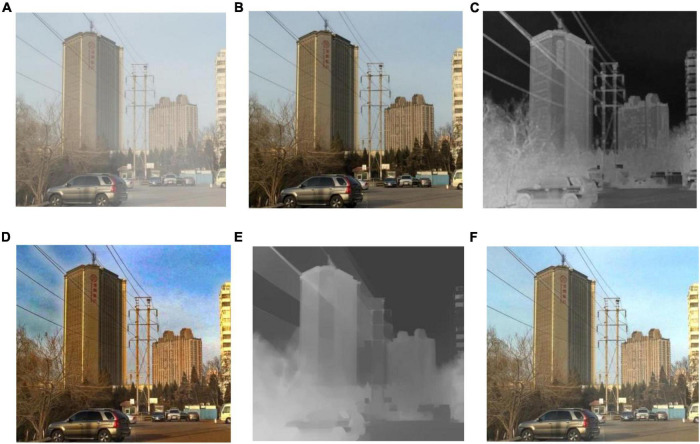
The effect of this method on transmission line transmittance optimization. **(A)** Hazy image; **(B)** ground truth **(C)** transmission of dark channel prior; **(D)** result of dark channel prior; **(E)** transmission of our method; **(F)** preliminary result of our method.

Figure 6 shows the comparison of the dark channel prior method and proposed method on transmission and recovered images. As shown in Figure 6C, when the transmission is estimated using the dark channel prior algorithm, the dark channel map contains some depth-independent details. And for thin overhead lines of electricity transmission lines, the range of the transmission map will be overestimated, resulting in halo artifacts near the overhead lines in the defogging results. As shown in Figure 6E, the proposed method can avoid overestimating the transmission of the overhead line, avoid halo artifacts near overhead line, and provide better transition at the junction of overhead line and sky. Comparing the method to the ground truth in Figure 6B, color saturation may also be avoided.

### 3.3. Detail sharpening

The transmission line is thin in size and difficult to observe, so it is necessary to enhance the details of the defogging image in order to better observe the electricity transmission line. The actual atmospheric scattering is multiple scattering, while the commonly used atmospheric scattering model is only one scattering, which will lead to the loss of details and blurred images in the defogging image. Therefore, it is necessary to sharpen the details of the defogging image. Image blur caused by multiple scattering is mainly related to two factors: visibility level and airlight level. The visibility level is related to detail, and the airlight level is related to depth (Gao et al., 2018). If the airlight level in a certain area of the image is high, the image details in that area will be smoother, so the degree of image sharpening is proportional to the airlight level. And the smoothness of the image details is poor when there is high visibility in a certain area, hence the degree of image sharpening is inversely related to the visibility level. The following is a definition of the sharpening coefficient:


(20) $$S\left(x,y\right)=S_{1}\left(x,y\right)\circ S_{2}\left(x,y\right)$$

In formula (20), *S*(*x, y*) represents the sharpening coefficient matrix. The function determined by the airlight level is represented by *S*_1_(*x, y*), while the function determined by the visibility level is represented by *S*_2_(*x, y*). *S*(*x, y*) means the multiplication of the corresponding elements of the *S*_1_(*x, y*) and *S*_2_(*x, y*) matrices.

Sigmoid function can satisfy the requirement that the airlight level is proportional to the sharpening coefficient, and the visibility level is inversely proportional to the sharpening coefficient. We use the cumulative distribution function as the constraint functions for the airlight level and visibility level. This function is a sigmoid function, expressed as follows:


(21) $$\phi \left(x\right)=\frac{1}{2}\left[1+erf\left(\frac{x}{\sqrt{2}}\right)\right]$$

The following formula is the error function *erf*(*x*):


(22) $$erf\left(x\right)=\frac{2}{\sqrt{\pi }}\int_{0}^{x}e^{-t^{2}}dt$$

The cumulative distribution function Φ(*x*) is an sigmoid function that increases monotonically with *x*. The cumulative distribution function can meet the requirement that the airlight level is proportional to the sharpening coefficient and the visibility level is inversely proportional to the sharpening coefficient. For the cumulative distribution function, it approaches 0 as *x* approaches −∞ and 1 as *x* approaches ∞, and the cumulative distribution function is a monotonically increasing function. If we add a minus sign to the cumulative distribution function, we get a monotonically decreasing function. Therefore, the cumulative distribution function can be used as the constraint function of airlight level and visibility level. As a result, the following definitions apply to the airlight level and visibility level constraints:


(23) $$S_{1}\left(x,y\right)=\frac{1}{2}\left\{1+erf\left[\frac{a\left(x,y\right)-a_{ave}}{\sqrt{2}k_{1}}\right]\right\}$$

(24) $$S_{2}\left(x,y\right)=1-\frac{1}{2}\left\{1+erf\left[\frac{C\left(x,y\right)-C_{ave}}{\sqrt{2}k_{2}}\right]\right\}$$

where *a*(*x, y*) denotes the airlight level, and *C*(*x, y*) represents the visibility level. *a*_*ave*_ represents the average value of the airlight level, *C*_*ave*_ represents the average value of the visibility level, and *k*_1_ and *k*_2_ are the slope control coefficients. The visibility level *C* has a relationship with the Weber brightness (Hautiere et al., 2008). The expression for visibility level *C* is as follows:


(25) $$C\left(x\right)=\frac{\Delta L\left(x,y\right)}{L_{b}\left(x,y\right)}=\frac{L_{t}\left(x,y\right)-L_{b}\left(x,y\right)}{L_{b}\left(x,y\right)}$$

In the above formula, Δ*L* is the brightness difference between the preliminary defogging result and the background image, *L*_*t*_ is the brightness of the preliminary defogging result, and *L*_*b*_ is the brightness of the image background. RGB space is the most commonly used color space, including three basic colors: red (R), green (G), and blue (B), while YCbCr is another color space, including luminance component (Y), blue chrominance component (Cb), and red chrominance component (Cr). To calculate the brightness difference, the image needs to be transferred from RGB space to YcbCr space. The preliminary defogging result is converted from RGB to YCbCr space, and the brightness component *L*_*t*_ is extracted, then the preliminary defogging result is low-pass filtered to produce *L*_*b*_.

The final enhancement result is as follows:


(26) $$J_{final}\left(x,y\right)=J\left(x,y\right)+\theta \cdot S\left(x,y\right)\circ T\left(x,y\right)$$

For formula (26), *J*_*final*_(*x*,*y*) is the final defogging result, *J*(*x*,*y*) is the preliminary defogging result, and the upper bound on the enhancement is constrained using *θ. T*(*x*,*y*) is the high-frequency value of the preliminary result *J*(*x*,*y*), which is obtained by Gaussian filtering on *J*(*x*,*y*) to prevent excessive enhancement of flat areas such as the sky. The preliminary defogging result *J* (*x, y*) in Formula (26) refers to the defogging result obtained by formula (8) after calculating the global atmospheric light *A* and transmission *t* of the input image *I* (*x*) through the methods of (Section “3.1 Global atmospheric light estimation”) and (Section “3.2 Transmission optimization”). That is, the fog-free image without detail sharpening.

**FIGURE 7:**
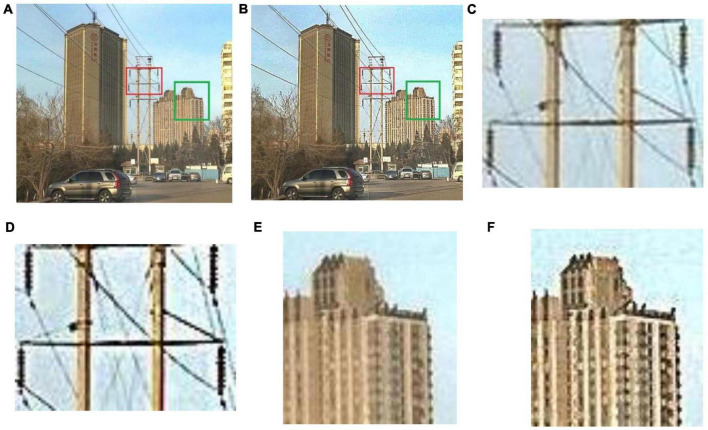
Comparison of local details between the two methods. **(A)** Preliminary defogging result without sharpening; **(B)** Final defogging result after sharpening; **(C)** Local detail of intermediate defogging result (insulators); **(D)** Local detail of final defogging result (insulators); **(E)** Local detail of intermediate defogging result (buildings); **(F)** Local detail of final defogging result (buildings).

The final defogging result after detail sharpening has more accurate local details. Figure 7 shows the comparison between the preliminary defogging results without sharpening and the final defogging results after sharpening. The details of the electricity transmission lines and insulators after sharpening are richer. According to Figure 7D, the electricity transmission line and insulator have more prominent image details after sharpening. In addition, the details of distant buildings have also become clearer. As shown in Figures 7E, F, the details of buildings in the image after detail sharpening are more prominent.

## 4. Experimental results and discussion

The efficiency of proposed defogging algorithm is examined through qualitative and quantitative comparisons with widely utilized defogging methods. We select foggy images with electricity transmission lines in the RESIDE dataset and compare with the methods of He et al. (2010), Fattal (2008), Meng et al. (2013), Tarel and Hautiere (2009), Ehsan et al. (2021), Berman and Avidan (2016), and Raikwar and Tapaswi (2020). The following parameters are selected for this study: *ξ* = 1.5, *λ* = 0.02, *ε* = 0.00001, *σ* = 0.2, *k*_1_ = 25, *k*_2_ = 0.01, *θ* = 3. The experimental platform is a 64-bit Windows 10 operating system laptop. The CPU is Inter(R) Core i7-11800 H and clocked at 2.30 GHz. The GPU is NVIDIA RTX3060. The computer memory is 40 GB. The software platform is MATLAB 2021b.

### 4.1. Qualitative comparison

We select 8 foggy images with electricity transmission lines or power towers from the OTS dataset in the RESIDE dataset with ground truth as research objects. We name images as Figures 1–8. The defogging results are shown in Figures 8–15. Note that the post-processing of the transmission of He’s method employs guided filter, rather than soft matting.

**FIGURE 8:**
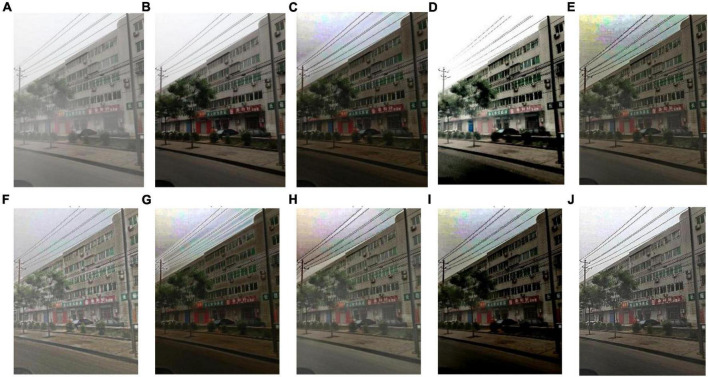
Experimental results of different methods for Figure 1: **(A)** input image; **(B)** ground truth; **(C)** dark channel prior; **(D)** Fattal et al.; **(E)** Meng et al.; **(F)** Tarel et al.; **(G)** Ehsan et al.; **(H)** Berman et al.; **(I)** Raikwar et al.; **(J)** proposed.

**FIGURE 9:**
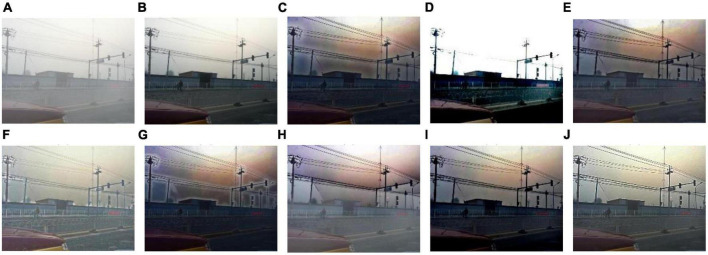
Experimental results of different methods for Figure 2: **(A)** input image; **(B)** ground truth; **(C)** dark channel prior; **(D)** Fattal et al.; **(E)** Meng et al.; **(F)** Tarel et al.; **(G)** Ehsan et al.; **(H)** Berman et al.; **(I)** Raikwar et al.; **(J)** proposed.

**FIGURE 10:**
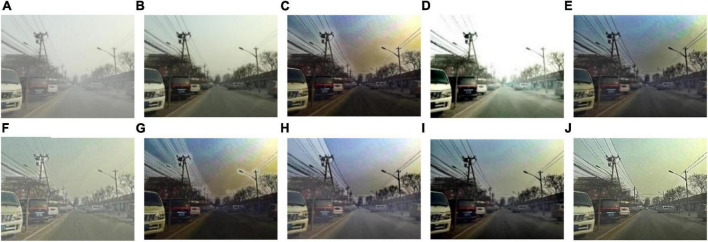
Experimental results of different methods for Figure 3: **(A)** input image; **(B)** ground truth; **(C)** dark channel prior; **(D)** Fattal et al.; **(E)** Meng et al.; **(F)** Tarel et al.; **(G)** Ehsan et al.; **(H)** Berman et al.; **(I)** Raikwar et al.; **(J)** proposed.

**FIGURE 11:**
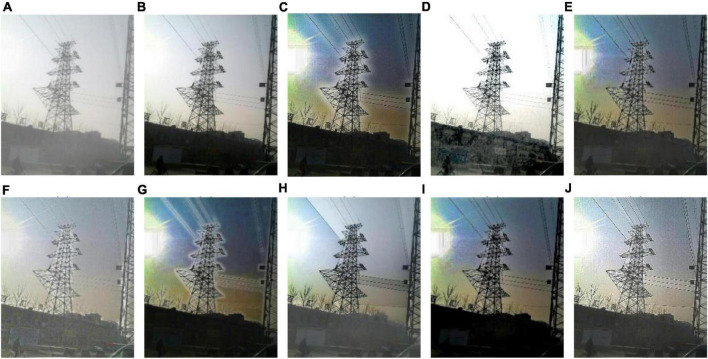
Experimental results of different methods for Figure 4: **(A)** input image; **(B)** ground truth; **(C)** dark channel prior; **(D)** Fattal et al.; **(E)** Meng et al.; **(F)** Tarel et al.; **(G)** Ehsan et al.; **(H)** Berman et al.; **(I)** Raikwar et al.; **(J)** proposed.

**FIGURE 12:**
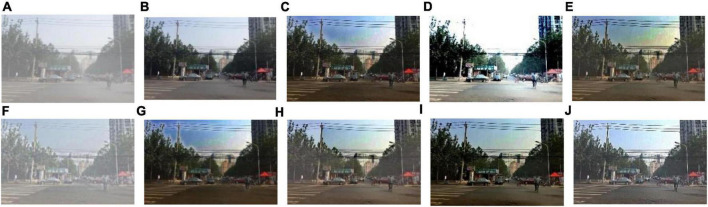
Experimental results of different methods for Figure 5: **(A)** input image; **(B)** ground truth; **(C)** dark channel prior; **(D)** Fattal et al.; **(E)** Meng et al.; **(F)** Tarel et al.; **(G)** Ehsan et al.; **(H)** Berman et al.; **(I)** Raikwar et al.; **(J)** proposed.

**FIGURE 13:**
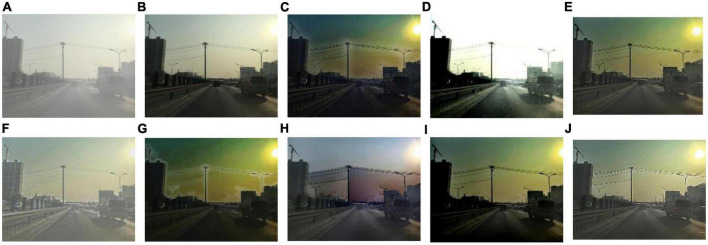
Experimental results of different methods for Figure 6: **(A)** input image; **(B)** ground truth; **(C)** dark channel prior; **(D)** Fattal et al.; **(E)** Meng et al.; **(F)** Tarel et al.; **(G)** Ehsan et al.; **(H)** Berman et al.; **(I)** Raikwar et al.; **(J)** proposed.

**FIGURE 14:**
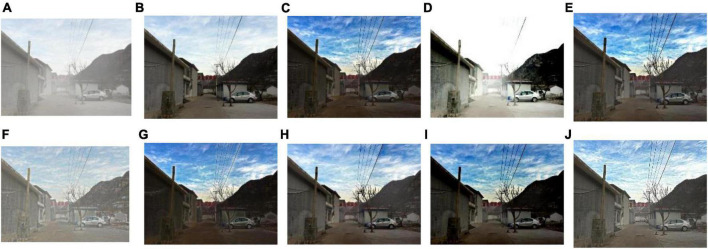
Experimental results of different methods for Figure 7: **(A)** input image; **(B)** ground truth; **(C)** dark channel prior; **(D)** Fattal et al.; **(E)** Meng et al.; **(F)** Tarel et al.; **(G)** Ehsan et al.; **(H)** Berman et al.; **(I)** Raikwar et al.; **(J)** proposed.

**FIGURE 15:**
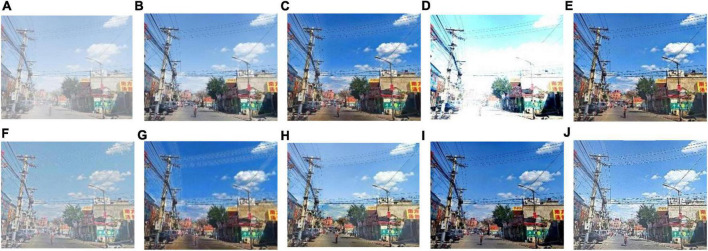
Experimental results of different methods for Figure 8: **(A)** input image; **(B)** ground truth; **(C)** dark channel prior; **(D)** Fattal et al.; **(E)** Meng et al.; **(E)** Tarel et al.; **(G)** Ehsan et al.; **(H)** Berman et al.; **(I)** Raikwar et al.; **(J)** proposed.

It can be seen from Figures 8–15 that dark channel prior and Meng’s method will over enhance the sky area, resulting in color deviation or over saturation of the sky area, while it is too dark for the non-sky area. Therefore, the fog removal image is very different from the ground truth. The reason for this is that the transmission is often underestimated when using these methods. At the same time, it is noted that when He’s method is used to defog the electricity transmission line, because the power tower and electricity transmission line are used as the foreground and the sky area is used as the background, there is a depth discontinuity between the foreground and the background, so there will be an obvious “halo” effect at the edge of the electricity transmission line, which is not conducive to observation. The “halo” phenomenon will lead to the transition area of color deviation between the image to be observed and the background, and produce abnormal colors of white or other colors around the image, affecting the observation of the image. This phenomenon is particularly obvious when dealing with Figures 2, 3, 4, 8.

For Fattal’s method, the sky area in the defogging image will be overexposed, resulting in chromatic aberration in the sky area. In addition, due to the tine size of the transmission line, the image of the electricity transmission line occupies a small proportion in the entire image, so the image information of the electricity transmission line in the defogging image is partially or completely lost, which is not conducive to the observation of the electricity transmission line. This phenomenon has a particularly obvious impact on the results of the Fattal’s method for defogging in Figures 5–8. For Tarel’s method, this method cannot completely remove fog, the image still retains a part of fog after defogging, and the overall image looks hazy with low saturation. For Ehsan’s method, there is a large chromatic aberration in the sky area, and there is a “halo” effect around the electricity transmission line, which is particularly evident in the defogging images of Figures 1, 3, 4, 6, 8. For Berman’s method, serious color distortion and color shift occur in the sky area of some defogging images, as shown in Figures 2, 3, 5, which are quite different from the ground truth. This is because this method needs to preset a gamma value for each image, and the most suitable gamma value for different images is an unknown quantity. If the best gamma value for each image is unknown, Berma recommends trying to set the default gamma value to 1. Therefore, Berman’s algorithm cannot satisfy all situations, and has limitations for practical use. Raikwar’s method can eliminate the “halo” phenomenon effectively, but it will still cause color saturation in the sky area, resulting in color shift. At the same time, the method also has low contrast in the non-sky area, which leads to darkness in the non-sky area of the defogging image and affects the observation of power towers on the ground.

For our algorithm, the color saturation of the image after defogging is moderate, the sky area is not over-saturated, and the ground area is not too low in brightness, the “halo” effect can be reduced at the same time. And the visual effect is most similar to the ground truth. In addition, because the proposed method sharpens and enhances the details of the defogging image, the power towers and electricity transmission lines in the defogging image have a clearer visual effect, retaining clearer outlines and more detailed details. It is convenient to observe electricity transmission lines and power towers. The qualitative study above shows that our method has better visual effects, and the fog removal effect is better and more realistic.

### 4.2. Quantitative comparison

The proposed defogging algorithm will be compared and analyzed with previous defogging algorithms utilizing various test metrics in the section on quantitative comparison. The following are the evaluation methods used in this study: peak signal-to-noise ratio (PSNR), information entropy, structural similarity index measurement (SSIM), mean squared error (MSE), universal quality index (UQI), average gradient (AG). According to whether there is a reference image, image evaluation methods can be divided into full reference image quality assessment and no reference image quality assessment. Full reference image quality assessment refers to comparing the difference between the image to be evaluated and the reference image when there is an ideal image as the reference image. No reference image quality assessment refers to directly calculating the visual quality of an image when there is no reference image. PSNR, SSIM, MSE, and UQI belong to full reference image quality assessment, while information entropy and AG belong to no reference image quality assessment. PSNR is the most commonly used image quality evaluation metric, which is an objective standard to measure the level of image distortion. The similarity between the fog removal image and the ground truth is directly proportional to the value of PSNR. A larger PSNR value means that the smaller the distortion of the defogging image and the better the defogging effect. SSIM is a measurement metric that objectively compares the brightness, contrast, and structure of two images to determine how similar they are to one another. The value of SSIM is a number in the range of 0 to 1, and the closer the value is to 1, the more closely the defogging images resemble the ground truth image. For an image, the average amount of information can be determined *via* the information entropy. The more details and richer colors of the image after defogging, the greater the information entropy. The UQI can reflect the structural similarity between two images. The larger the value of UQI, the closer the two images are. The AG is related to the changing characteristics of the image detail texture and reflects the sharpness. The clearer the image, the higher the value of AG. Note that SSIM and UQI belong to the full reference image quality assessment and need to be compared with the reference image when calculating. Therefore, we selected the ground truth of OTS data set as the reference image, compared the defogging images obtained by different methods with the ground truth, and obtained the evaluation results. Similarly, PSNR and MSE also chose ground truth as the reference image.

Tables 1–6 show the results of evaluation metrics obtained when different defogging algorithms are adopted in Figures 1–8. For each row in the table, the bold value represents that the evaluation metric can obtain the optimal result when the defogging algorithm corresponding to the value is adopted for the image.

**TABLE 1:** Comparison of peak signal-to-noise ratio (PSNR) of the defogging images.

Figure	He	Fatal	Meng	Tarel	Ehsan	Berman	Raikwar	Our
I	17.4379	16.3903	15.4995	12.5983	16.2771	14.9417	13.8952	**19.82**
II	12.4694	14.655	12.1856	15.3734	12.0424	13.0669	12.7189	**18.5631**
III	14.8967	13.5464	13.5908	14.4461	13.599	16.1208	14.8689	**20.7617**
IV	11.8169	14.5616	11.985	14.6685	11.2372	15.6598	11.4271	**18.2309**
V	16.8005	12.7247	16.6877	10.9627	15.965	18.8939	16.0547	**19.2684**
VI	11.6662	12.5876	13.9475	13.1485	11.4975	15.1161	13.0413	**17.9578**
VII	14.7942	12.2326	15.6319	12.8542	14.4614	19.006	13.8794	**19.6325**
VIII	16.0436	11.5157	16.8198	16.5496	15.0584	20.0116	14.0607	**20.1302**
Average	14.4907	13.5267	14.5435	13.8252	13.7673	16.6021	13.7433	**19.2956**

**TABLE 2:** Comparison of structural similarity index measurement (SSIM) of the defogging images.

Figure	He	Fatal	Meng	Tarel	Ehsan	Berman	Raikwar	Our
I	**0.7908**	0.6541	0.7542	0.6952	0.7373	0.7186	0.5402	0.7857
II	0.6939	0.4905	0.6952	0.7566	0.6577	0.652	0.5856	**0.7621**
III	0.6798	0.6411	0.6309	**0.8271**	0.6425	0.5822	0.6662	0.8238
IV	0.706	0.6852	0.6622	**0.7349**	0.7057	0.7057	0.7057	0.6986
V	0.7357	0.487	0.6886	0.7378	0.7095	0.7411	0.6692	**0.785**
VI	0.6548	0.5038	0.795	0.7476	0.6958	0.5672	0.5852	**0.8185**
VII	0.7135	0.4965	0.7285	0.7521	0.6884	**0.8242**	0.6088	0.7868
VIII	0.8501	0.5455	0.8467	0.8562	0.8287	**0.9023**	0.7999	0.862
Average	0.7281	0.563	0.7252	0.7634	0.7082	0.7117	0.6451	**0.7903**

**TABLE 3:** Comparison of information entropy of the defogging images.

Figure	He	Fatal	Meng	Tarel	Ehsan	Berman	Raikwar	Our
I	7.1639	6.586	7.3179	7.0902	7.0475	7.2592	6.9901	**7.3549**
II	7.4548	4.1126	7.4348	7.3702	7.3647	7.3263	7.3466	**7.5985**
III	7.5324	5.6041	7.4242	6.8307	7.3969	**7.5966**	7.5963	7.4889
IV	7.5499	5.5866	7.4739	7.2516	7.438	**7.7022**	6.9906	7.5764
V	7.3755	6.5764	7.3821	6.5202	7.2797	7.3142	**7.5267**	7.4763
VI	7.1002	5.2216	7.246	6.9646	7.1061	7.3705	7.1291	**7.4727**
VII	7.4989	5.6138	7.5771	6.9565	7.3666	7.5943	7.5273	**7.6219**
VIII	7.5316	5.6522	7.5435	6.678	7.4648	7.4519	7.5322	**7.6213**
Average	7.4009	5.6192	7.4249	6.9578	7.308	7.4519	7.3299	**7.5264**

**TABLE 4:** Comparison of mean squared error (MSE) of the defogging images.

Figure	He	Fatal	Meng	Tarel	Ehsan	Berman	Raikwar	Our
I	1172.9902	1492.9522	1832.8653	3574.8292	1532.4007	2084.0716	2651.9413	**677.7674**
II	3682.4637	2226.287	3931.1625	1886.8427	4062.9096	3209.1541	3476.8593	**905.2525**
III	2105.7584	2873.7249	2844.4478	2335.9851	2839.0908	1588.5627	2119.313	**545.6503**
IV	4279.4657	2274.6594	4117.0035	2219.371	4890.5339	2256.9768	4681.292	**977.2104**
V	1358.4117	3472.2773	1394.1472	5209.7195	1646.554	1646.554	1612.9123	**769.5488**
VI	4430.6094	3583.5926	2620.1912	3149.4204	4606.1166	2002.0382	3228.1386	**1040.6388**
VII	2156.0511	3888.8094	1777.8414	3370.2747	2327.7683	817.48	2661.5958	**707.6773**
VIII	1617.0443	4586.8511	1352.3898	1439.1915	2028.7847	648.512	2552.7462	**631.0411**
Average	2600.3493	3049.8942	2483.7561	2898.2043	2991.7698	1781.6687	2873.0998	**781.8483**

**TABLE 5:** Comparison of universal quality index (UQI) of the defogging images.

Figure	He	Fatal	Meng	Tarel	Ehsan	Berman	Raikwar	Our
I	0.8314	0.7429	0.8439	0.6734	0.7999	0.7679	0.5632	**0.8665**
II	0.726	0.6864	0.8223	0.7922	0.7022	0.7529	0.6101	**0.9369**
III	0.8077	0.8525	0.8725	0.8331	0.7643	0.902	0.7829	**0.9638**
IV	0.7683	0.8261	0.7391	0.7753	0.7278	0.7912	0.5586	**0.8571**
V	0.8791	0.7446	0.9149	0.6979	0.8565	0.898	0.7825	**0.9234**
VI	0.6906	0.7417	0.8542	0.7017	0.7494	0.8552	0.6078	**0.883**
VII	0.8367	0.7472	0.8857	0.7334	0.8224	**0.9041**	0.6716	0.8886
VIII	0.9238	0.8622	0.9433	0.9232	0.9023	0.9645	0.8122	**0.9781**
Average	0.808	0.7755	0.8595	0.7663	0.7906	0.8545	0.6736	**0.9122**

**TABLE 6:** Comparison of average gradient (AG) of the defogging images.

Figure	He	Fatal	Meng	Tarel	Ehsan	Berman	Raikwar	Our
I	6.2546	9.3965	7.9195	7.2134	6.1852	7.6195	7.6144	**10.391**
II	4.8603	4.7456	6.3885	5.1114	4.6492	5.3087	5.7916	**8.4631**
III	7.138	7.2709	8.193	5.6889	7.006	8.3407	8.1677	**10.0117**
IV	8.6812	8.9836	10.0807	8.1328	8.8091	9.1037	10.1014	**12.2277**
V	8.215	12.1108	9.1919	6.1002	7.9448	8.7118	9.3035	**12.5756**
VI	2.7866	3.4014	2.8994	2.9219	2.5547	3.1448	2.8588	**4.902**
VII	5.4091	4.6841	6.4485	4.4309	5.0904	5.7151	6.1237	**7.6858**
VIII	11.7685	10.2167	14.1335	7.9979	11.8835	12.1639	13.4144	**17.8921**
Average	6.8892	7.6012	8.1569	5.9497	6.7654	7.5135	7.9219	**10.5186**

As shown in Tables 1–6, for PSNR, MSE and AG, compared with other comparison algorithms, the algorithm proposed in this paper achieves the best effect for each image, and obviously the average evaluation results also achieve the best effect. For SSIM, information entropy and UQI, the proposed method achieves the highest or relatively high performance on single image metrics, respectively, and the best performance on average score. For SSIM, the average value obtained by the method proposed in this paper is 0.7903, which is 3.4% higher than that of Tarel, the second highest ranking method, and 28.76% higher than that of Fattal, the lowest ranking method. For information entropy, the method proposed in this paper achieves an average value of 7.5264, which is 0.99% higher than Berman’s method with the second highest ranking and 25.34% higher than Fattal’s method with the lowest ranking. For UQI, the method proposed in this paper achieves an average value of 0.9122, which is 5.78% higher than that of Meng, the second highest ranking method, and 26.16% higher than that of Raikwar, the lowest ranking method. Quantitative analysis show s that the image obtained by using the proposed method has better structure similarity, rich information content, better color restoration and clarity. As a result, the proposed method has good visual effect.

For the evaluation results of a single defogging image, Berman’s method and Raikwar’s method will cause color shift and over-saturation in the sky area, making the sky area more yellow or blue. And the information entropy is an indicator that reflects the richness of the color, so the information entropy is sometimes higher when using Berman’s method and Raikwar’s method. At the same time, Berman’s method requires a gamma value to be set in advance, so the application scenarios are limited. Although Tarel’s method is used for some images to obtain the best SSIM, Tarel’s method cannot completely remove the fog, and the details of electricity transmission lines in the defogging image are not obvious, which is not conducive to observation.

In general, this method can enhance image details, avoid image distortion and color offset, and has a good defogging effect.

## 5. Conclusion

In this study, we propose an image defogging algorithm for power towers and electricity transmission lines in video monitoring system. First of all, in view of the statistical law that most of the outdoor electricity transmission line images have a sky area in the upper part of the image, the proposed algorithm uses an improved quadtree segmentation algorithm to find the global atmospheric light, then locates the global atmospheric light in the sky area containing the electricity transmission line, preventing the white or bright objects on the ground interfere with the calculation of global atmospheric light. Second, to solve the “halo” effect when the transmission is computed by the dark channel prior, the algorithm in this paper introduces the concept of dark pixels, and uses superpixel segmentation to locate the dark pixels and use a fidelity function to compute the transmission. Finally, in view of the problem that the size of outdoor electricity transmission lines is tiny and unsuitable for observation, this paper introduces a detail enhancement post-processing based on visibility level and air light level to enhance the details of defogging images. We assess the efficacy of the proposed method by quantitatively and qualitatively assessing defogging images of power towers and transmission lines that were acquired using various methods. The results of the experiment proved that the defogging images restored by suggested algorithm have better detail level, structural similarity and color reproduction, and can effectively remove fog, which is superior to existing algorithms. In addition, the algorithm proposed in this paper can not only be used in power system online monitoring, but also can be extended to community monitoring, UAV monitoring, automatic driving, industrial production, Internet of Things and other fields, with broad application space. In the further work, we are going to do research on image defogging combined with dark image en-hancement to expand the application range of the algorithm.

## Data availability statement

Publicly available datasets were analyzed in this study. This data can be found here: https://github.com/h4nwei/MEF-SSIMd.

## Author contributions

MZ and MG: conceptualization. ZS: methodology. JY: software, formal analysis, and writing—original draft preparation. JY and MG: validation and data curation. CY and ZJ: investigation. ZJ: resources. MG: writing—review and editing. YH: visualization and supervision. MZ and WC: project administration. MZ: funding acquisition. All authors have read and agreed to the published version of the manuscript.
